# Meta-analysis suggests the microbiome responds to Evolve and Resequence experiments in *Drosophila melanogaster*

**DOI:** 10.1186/s12866-021-02168-4

**Published:** 2021-04-09

**Authors:** Lucas P. Henry, Julien F. Ayroles

**Affiliations:** 1 Department of Ecology & Evolutionary Biology, 150 Carl Icahn Laboratory, Princeton University, Princeton, NJ 08544 USA; 2 Lewis-Sigler Institute for Integrative Genomics, Princeton University, Princeton, NJ 08544 USA

**Keywords:** Experimental evolution, *Drosophila melanogaster*, Microbiome

## Abstract

**Background:**

Experimental evolution has a long history of uncovering fundamental insights into evolutionary processes, but has largely neglected one underappreciated component--the microbiome. As eukaryotic hosts evolve, the microbiome may also respond to selection. However, the microbial contribution to host evolution remains poorly understood. Here, we re-analyzed genomic data to characterize the metagenomes from ten Evolve and Resequence (E&R) experiments in *Drosophila melanogaster* to determine how the microbiome changed in response to host selection.

**Results:**

Bacterial diversity was significantly different in 5/10 studies, primarily in traits associated with metabolism or immunity. Duration of selection did not significantly influence bacterial diversity, highlighting the importance of associations with specific host traits.

**Conclusions:**

Our genomic re-analysis suggests the microbiome often responds to host selection; thus, the microbiome may contribute to the response of *Drosophila* in E&R experiments. We outline important considerations for incorporating the microbiome into E&R experiments. The E&R approach may provide critical insights into host-microbiome interactions and fundamental insight into the genomic basis of adaptation.

**Supplementary Information:**

The online version contains supplementary material available at 10.1186/s12866-021-02168-4.

## Background

The microbiome has emerged as a key modulator of many organismal phenotypes [1–3]. While many studies show the impact of the microbiome on host phenotypes, the evolutionary implications remain enigmatic [4–6]. The microbiome may contribute to host evolution in unique ways. First, large effective population sizes and rapid generation times may enable microbes to evolve more rapidly than hosts [7]. Second, the microbiome likely encodes distinct genes compared to the host genome, potentially expanding the genomic reservoir to enable adaptation to diverse selective pressures [3, 8, 9]. If hosts can leverage this microbial evolution, then the microbiome may alter host evolution.

Experimental evolution is a powerful tool to study the basis of adaptation, but remains underutilized in the study of host-microbiome evolution [6, 10, 11]. One particularly well-suited class of these studies is Evolve and Resequence (E&R) experiments [12–14]. E&R experiments build on the long history of using artificial selection in evolutionary biology by incorporating new advances in sequencing technologies to measure the genomic responses to selection. E&R experiments are commonly performed in microbes like *E. coli* or yeast, as well as eukaryotes like *Drosophila* [13]. In general, E&R experiments begin with large outbred populations. The population is reared under a particular selective regime. The selective regime can take many forms, ranging from threshold selection (e.g., egg size) or general survival under some sort of stressor (e.g., low nutrition diets). In parallel, to control for genetic drift, control populations are maintained in a benign (i.e., non-selective) environment. After a number of generations, the control and evolved populations are sequenced to identify regions of the genomes associated with the response to selection. For flies and other eukaryotic hosts, selection is explicitly applied to host populations, but may also act upon the microbiome. When the microbiome influences host phenotypic variation, microbial variation may also affect the response to selection in hosts. Thus, the underappreciated interplay between host and microbial variation has the potential to complicate the interpretation of selection responses based strictly on host genetic variation.

Microbes may be underappreciated drivers of host phenotypic variation. For example, *Wolbachia* infection can rescue deleterious phenotypes in homozygous mutant *Drosophila* lines [15–17]*.* Body color in aphids is partially determined by *Rickettsia* secondary symbionts [18]. These phenotypic effects are not limited to single microbial species, but also include more complex microbiomes. In cows, the microbiome explained 13% of methane emissions [19] and 26–42% of fatty acid composition of milk [20]. The microbiome also explained 33% of weight gain in pigs [21]. For both pigs and cows, the microbiome contributed almost as much to traits as host genetics. These examples suggest that the microbiome in many host taxa is an important determinant in host phenotypes, and, in turn, may shape the selection response for hosts. E&R experiments may thus be missing a substantial component that shapes the host evolutionary response.

Here, we analyzed the metagenomes from 10 E&R experiments in *Drosophila melanogaster.* Many phenotypes in *D. melanogaster* are responsive to microbial variation, including developmental, metabolic, and immunological traits [22–24]. Furthermore, E&R experiments in *D. melanogaster* capture the evolutionary response to a wide range of different selective pressures, ranging from life history to nutritional to pathogen challenges (Table 1). Thus, E&R experiments in *D. melanogaster* provide a unique opportunity to study how the microbiome responds to host selection. Our goal here is to explore these publicly available data and using meta-analysis and characterize patterns in the metagenomes of these experiments. This meta-analysis allowed us to identify common patterns in the response of the microbiome to host selection. We use these observations to highlight potential future directions for which the powerful E&R approach is uniquely suited to identify signatures of selection in host-microbiome evolution.

**Table 1:** Evolve & Resequence studies analyzed

Pressure	Evolved Phenotype	Duration of Selection (generations)	***Wolbachia*** (% reads, min-max)	Experimental design (D: diet, S: sequencing)
Accelerated development [25]	Flies developed from egg to adult 20% faster than control	605	Infected (0.01–1.40%)	D: Banana, corn syrup, agar. S: 25 females (age not reported) pooled from each line; 4 control and 4 evolved.
Delayed reproduction [26]	Age of reproduction increased from 28 to 40 days	50	Uninfected (0%)	D: Not specified. S: 100 females (age not reported) pooled from each line; 18 control and 18 evolved.
Increased lifespan [27]	Median lifespan was increased from 4 weeks to 7–8 weeks	48	Uninfected (0%)	D: Agar, yeast, sugar, oatmeal. S: 250 males + 250 females (age not reported) pooled from each line; 3 control and 3 evolved.
Egg size [28]	Egg size was selected ~ 20% larger and smaller eggs	16	Infected (0.02–0.11%)	D: Not specified. S: 100 females (age not reported) pooled from each line; 3 control, 3 small, 3 large.
Desiccation resistance [29]	Desiccation resistance (hrs until 80% mortality) increased 70–80%	48	Infected (70.2–89.7%)	D: Yeast, cornmeal, agar. S: 100 females (age not reported) pooled from each line; 3 control and 3 evolved.
Fluctuating temperature [30]	Survival under fluctuating temps 18–28 °C daily	37	Infected (48.7–75.1%)	D: Standard media. S: 500 females (~ 7 days old) pooled for each line at different time points; beginning, middle, and end; 3 control and 3 evolved--only compared beginning and end.
Salt + cadmium resistance [31]	Survival in constant, spatially, temporally varying salt and/or cadmium	42	Infected (0.01–0.03%)	D: Yeast, cornmeal, sugar agar. Diet differed between control and evolved. S: 70 females (age not reported) pooled from each line; 3 control lines and 5 lines for each selection pressure.
Starvation resistance [32]	Starvation resistance (hrs to death w/o food) increased ~ 25%	83	Infected (53.0–78.4%)	D: Not specified. S: 100 females (4 days old) pooled for each line; 3 control and 3 evolved lines.
Parasitoid resistance [33]	Resistance to parasitoid increased from 20 to 50%	5	Uninfected (0%)	D: Not specified. S: 50 females (~ 5 h old) pooled from each line; 16 control and 16 evolved lines.
Viral resistance [34]	Resistance to Drosophila C virus increased from 25 to 75%	20	Infected (95.1–98.1%)	D: Standard cornmeal-agar. S: 200 individuals (age not reported) pooled from each line; 4 control, 4 procedure control, 4 evolved.

## Results

The 10 E&R experiments analyzed for metagenomes ranged in a variety of selective pressures (see Table 1 for full description)--from life history (accelerated development [25], delayed reproduction [26], increased lifespan [27], egg size [28]) to abiotic pressures (desiccation resistance [29], fluctuating temperature [30], salt and heavy metal resistance [31]) to biotic pressures (starvation resistance [32], parasitoid resistance [33], viral resistance [34]). All experiments had replicated control and evolved populations, although replication varied from as few as three to as many as 18 (Table 1). Given the importance of the diet in shaping microbial variation, we examined the reported characteristics of the diet from each study. Importantly, for 9/10 studies, the reported diets did not differ between control and evolved populations (Table 1). Only for the cadmium and salt resistance study [31] were diets different for the entire lifespan between control and evolved populations. The starvation resistance study [32] exerted starvation on adults for 4 days, and then flies that survived were returned to a standard diet to propagate the next generation. While the diets may have varied between studies, only 5/10 studies described the diet (Table 1). The lack of consistent dietary reporting is a major challenge for *Drosophila*-microbiome studies [35]. As the majority of these studies do not report specific dietary information, we are unable to explore the effects of diet across E&Rs in this analysis.

**Fig. 1:**
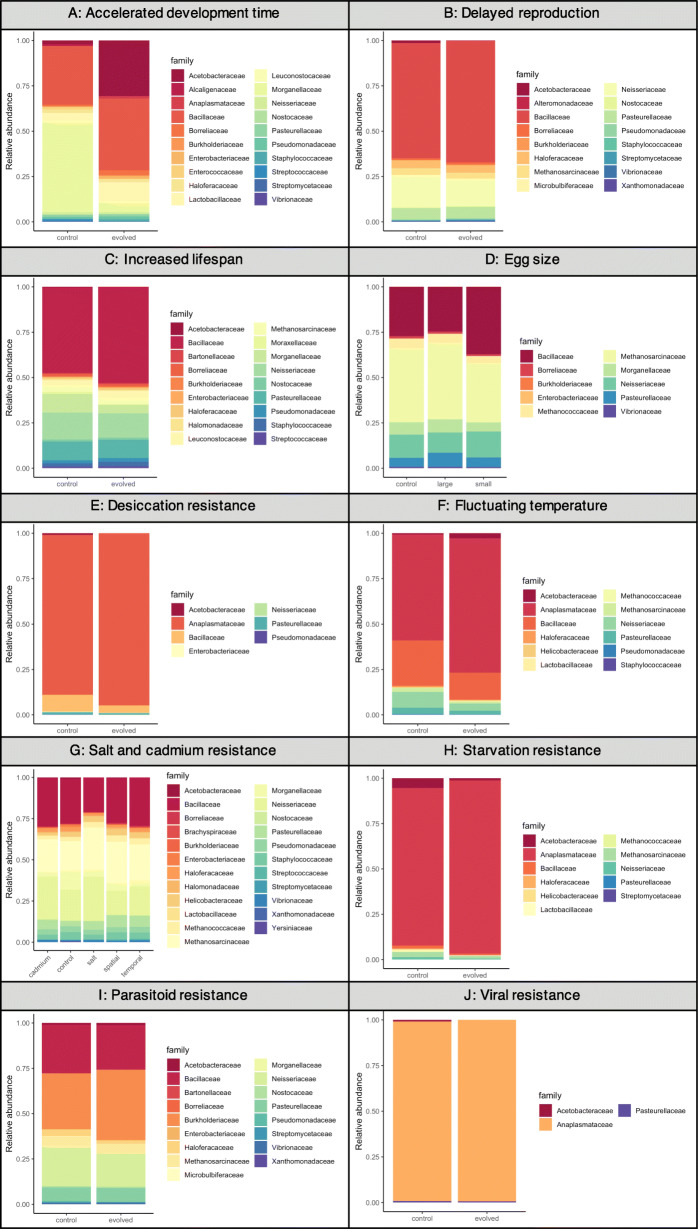
Relative abundance for bacterial families from the 10 E&R experiments. Each experiment was grouped separately; the colors represent different bacterial families in each. Only bacteria that comprised > 1% of total reads were visualized

**Fig. 2:**
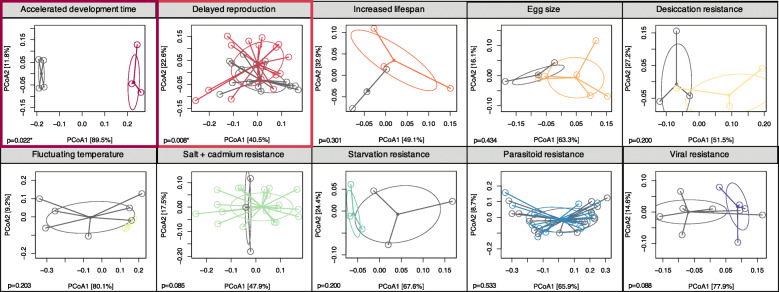
PCoA plots for beta-diversity using Jaccard similarity for the 10 E&R experiments show the majority of control and evolved populations harbor similar bacterial families. Each experiment was grouped separately; grey color represents control populations, and the colored points represent evolved populations. Each sequenced pool is shown as a point with lines connecting to the centroid based on PCoA clustering. The two studies (accelerated development time and delayed reproduction) with significantly divergent microbiomes between control and evolved populations are outlined in colored boxes. *P*-values are shown for all studies

**Fig. 3:**
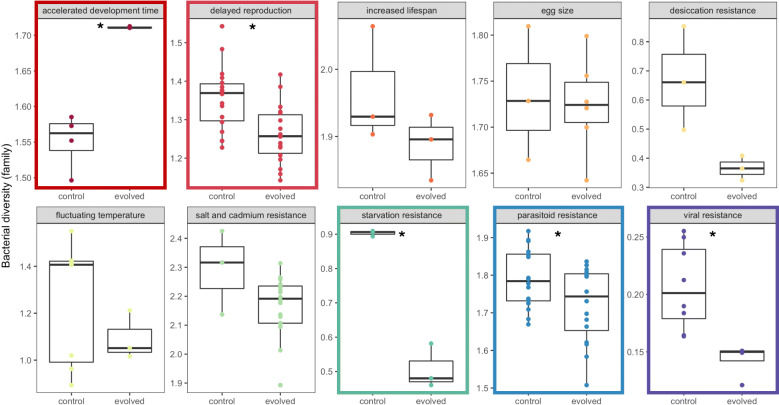
Bacterial diversity between control and evolve populations in 10 E&R experiments. 5/10 experiments had significantly different bacterial diversity (denoted with the colored outline and asterisk). Bacterial diversity was calculated at the family level using Shannon diversity metric. Comparisons between control and evolved populations were within each experiment. Each point represents a pool of sequenced flies showing replication within control and evolved groups, and the details of how many flies/experiments are described in Table 1. Color represents each experiment

Because each experiment has replicated control and evolved populations, we compared microbiomes within each experiment. In the E&R context, control populations represent the standing genetic variation from which selection proceeds. Thus, by comparing control and evolved populations within experiment, the effects of many different factors (e.g., local laboratory environment, different diets, different fly populations) are controlled for in our analysis. For each experiment, bacterial families were differentially abundant in control and evolved populations (Fig. 1; Supp. Figs 1-10 for individual replicates for each experiment). Control and evolved populations tended to harbor similar bacterial families across replicates, within each experiment, as measured through beta-diversity (Jaccard similarity; Fig. 2, Table 2). Only in two experiments did control and evolved populations differ in community membership--accelerated development time and delayed reproduction. Bacterial alpha-diversity frequently responded to experimental evolution (Fig. 3). Evolved populations often exhibited reduced levels of bacterial diversity (4/10 studies), though in one case (accelerated development time) bacterial diversity increased (Table 3 for statistical summary). Taken together, the microbiome frequently shifts in response to host selection (i.e., differences in alpha-diversity), but does not necessarily gain different microbes (i.e., no difference in beta-diversity).

**Table 2:** Summary statistics for beta-diversity (Jaccard similarity)

**Accelerated development time**						
	Df	SumsOfSqs	MeanSqs	F.Model	R^2^	Pr(>F)
group	1	0.30394	0.303937	40.862	0.89098	0.022*
Residuals	5	0.03719	0.007438		0.10902	
Total	6	0.34113			1	
**Delayed reproduction**						
	Df	SumsOfSqs	MeanSqs	F.Model	R^2^	Pr(>F)
group	1	0.09902	0.099022	3.9017	0.10294	0.008**
Residuals	34	0.86288	0.025379		0.89706	
Total	35	0.9619			1	
**Increased lifespan**						
	Df	Sum Sq	Mean Sq	F	N.Perm	Pr(>F)
Groups	1	0.0031624	0.0031624	1.846	719	0.3014
Residuals	4	0.0068525	0.0017131			
Egg size						
	Df	Sum Sq	Mean Sq	F	N.Perm	Pr(>F)
Groups	1	0.0020672	0.0020672	0.7579	999	0.434
Residuals	7	0.0190935	0.0027276			
**Desiccation resistance**						
	Df	SumsOfSqs	MeanSqs	F.Model	R^2^	Pr(>F)
group	1	0.044141	0.044141	1.8976	0.32176	0.2
Residuals	4	0.093047	0.023262		0.67824	
Total	5	0.137188			1	
**Fluctuating temperature**						
	Df	SumsOfSqs	MeanSqs	F.Model	R^2^	Pr(>F)
group	1	0.11007	0.11008	1.9823	0.19858	0.203
Residuals	8	0.44424	0.05553		0.80142	
Total	9	0.55432			1	
**Salt + cadmium resistance**						
	Df	SumsOfSqs	MeanSqs	F.Model	R^2^	Pr(>F)
group	1	0.05652	0.056519	2.1164	0.09155	0.085
Residuals	21	0.56081	0.026705		0.90845	
Total	22	0.61733			1	
**Starvation resistance**						
	Df	SumsOfSqs	MeanSqs	F.Model	R^2^	Pr(>F)
group	1	0.019872	0.019872	2.0937	0.34359	0.2
Residuals	4	0.037964	0.009491		0.65641	
Total	5	0.057836			1	
**Parasitoid resistance**						
	Df	SumsOfSqs	MeanSqs	F.Model	R2^2^	Pr(>F)
group	1	0.04295	0.04295	0.64716	0.02112	0.533
Residuals	30	1.99099	0.066366		0.97888	
Total	31	2.03394			1	
**Viral resistance**						
	Df	SumsOfSqs	MeanSqs	F.Model	R2^2^	Pr(>F)
group	1	0.0384	0.0384	2.673	0.21092	0.088
Residuals	10	0.14366	0.014366		0.78908	
Total	11	0.18206			1

**Table 3:** Statistical differences between control and evolved microbiomes

Pressure	test stat	df	significance
accelerated development	t = −8.116	df = 3.009	*p*-value = 0.004
delayed reproduction	t = 3.558	df = 33.652	*p*-value = 0.001
increased lifespan	t = 1.364	df = 3.172	*p*-value = 0.261
egg size	t = 0.213	df = 3.105	*p*-value = 0.845
desiccation resistance	t = 2.883	df = 2.224	*p*-value = 0.090
fluctuating temperature	t = 1.238	df = 7.996	*p*-value = 0.251
salt and cadmium resistance	t = 1.431	df = 2.287	*p*-value = 0.274
starvation resistance	t = 10.448	df = 2.065	*p*-value = 0.008
parasitoid resistance	t = 2.179	df = 28.394	*p*-value = 0.038
viral resistance	t = 4.265	df = 9.829	*p*-value = 0.002

**Fig. 4:**
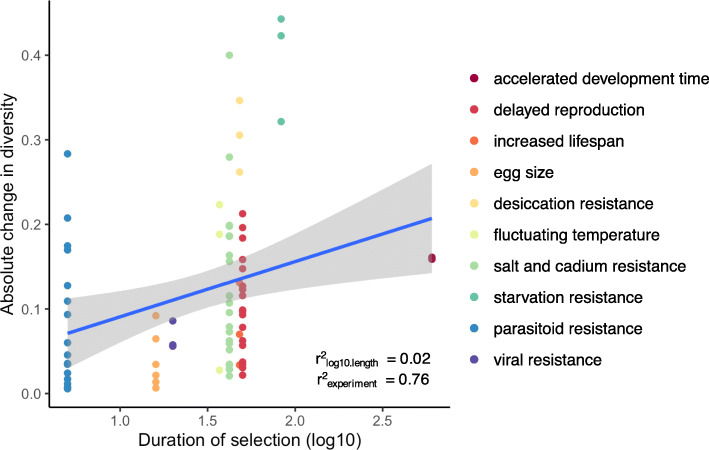
Evolved bacterial diversity was not significantly correlated with duration of selection after controlling for differences between experiments as random effects (*r* = 0.05, *p* = 0.649). Random effects (experiment) explained 76% of variation (see Supp. Fig. 11). Generations of selection range from 5 generations (parasitoid resistance) to > 500 generations (accelerated development). Each point represents the difference between average control diversity and each pool of evolved flies for each experiment. Points are colored by experiment

Because the number of generations varied across E&R experiments (from 5 to 605 *Drosophila* generations; see Table 1), we also tested if change in microbial diversity was correlated with duration of host selection. One hypothesis is that shorter selection experiments provide less opportunity for the microbiome to change, while longer selection provides more opportunity for increased microbial change. The change in microbial diversity was not correlated with duration of selection after controlling for each study as a random effect (Fig. 4, *r* = 0.05, *p* = 0.649). The specific nature of the selective pressure appears to be more important in driving changes in the evolving microbiome as experiment explained 76% of variance in our model (Table 4). For example, the evolved microbiome in the starvation resistance experiment exhibited the greatest change in bacterial diversity. This may not be surprising given that the *Drosophila* microbiome has been shown to be tightly linked to the regulation of metabolic networks [22]. For other traits, like egg size, the microbiome did not significantly respond to experimental evolution. This analysis suggests that the effect of selection on microbiome is likely trait specific.

**Table 4:** Summary statistics of mixed model to assess the relationship between duration of selection and change in diversity. Random effect was modeled as experiment (i.e., accelerated development time, starvation resistance, etc.)

***Fixed effects***			
	Estimate (std. error)	T-value	Pr (> | t |)
Intercept	−0.185 (0.162)	−1.137	0.291
log10.length	0.046 (0.097)	0.473	0.649
***Random effects***			
	Variance	Std. deviation	
Experiment	0.0225	0.1500	
Residual	0.007	0.084	
***Summary***			
Observations		79	
Log Likelihood		66.128	
Akaike Inf. Crit.		−124.256	
Bayesian Inf. Crit.		−114.779	

**Fig. 5:**
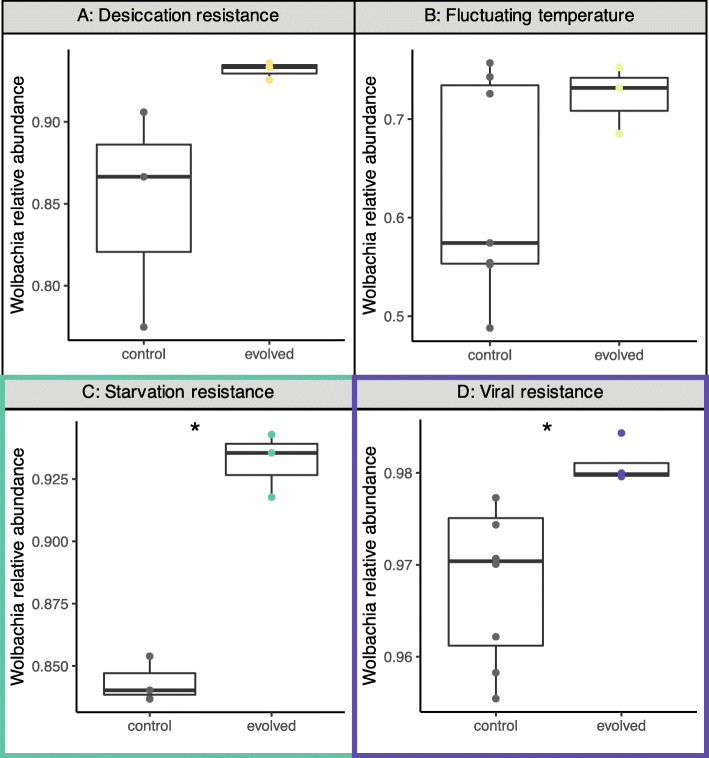
*Wolbachia* relative abundance tended to increase in evolved populations for four studies, but was only statistically significant in starvation resistance and viral resistance (denoted with colored box and asterisk). Comparisons were made within each study between control and evolved populations. Each point represents a pool of flies

We note that, in seven out of ten studies, flies were infected with *Wolbachia* (Table 1). *Wolbachia* was < 2% of reads for three studies, but 48–98% for the other four studies. To better understand the association between *Wolbachia* and the microbial response to selection, we focused on these four studies with high relative abundance (Fig. 5). *Wolbachia* was significantly more abundant for evolved populations in starvation and viral resistance, though also tended to increase for desiccation resistance and fluctuating temperatures (Table 5 for statistical summary). Taken together, these results highlight how *Wolbachia* may underlie some of the significant changes to diversity that occur in evolved populations.

**Table 5:** Statistical differences in *Wolbachia* relative abundance between control (C) and evolved (E) populations

Experiment	C min	C max	C avg	E min	E max	E avg
**Desiccation resistance**						
Relative abundance	0.775	0.906	0.849	0.926	0.936	0.932
Kruskal-Wallis X^2^ = 3.8571, df = 1, *p*-value = 0.04953						
**Fluctuating temperature**						
Relative abundance	0.488	0.757	0.628	0.685	0.752	0.723
Kruskal-Wallis X^2^ = 1.0519, df = 1, *p*-value = 0.3051						
**Starvation resistance**						
Relative abundance	0.837	0.854	0.844	0.918	0.943	0.932
Kruskal-Wallis X^2^ = 3.8571, df = 1, *p*-value = 0.04953						
**Viral resistance**						
Relative abundance	0.955	0.977	0.968	0.98	0.984	0.981
Kruskal-Wallis X^2^ = 7.3846, df = 1, *p*-value = 0.006578						

## Discussion

To our knowledge, this is the first systematic examination of the microbiome in E&R experiments in *D. melanogaster*. Given the many fundamental insights gained from *Drosophila* in E&R experiments [13], our results here uncover another layer of variation previously unexplored--the microbiome. The microbiome changed under some selective pressures, while it was unaffected by others (Figs. 1 and 3). Pressures closely linked to metabolic processes, like starvation resistance or development time, or immunity affected microbial diversity the most. In *Drosophila*, bacterial genes that increase glucose assimilation and fat storage are necessary for bacterial establishment in the host gut, suggesting that hosts select for bacteria to enhance metabolism [36–38]. Other pressures, like selection for increased lifespan, egg size, or abiotic stressors (e.g temperature and heavy metals), did not substantially impact microbial diversity (Fig. 3). It is not surprising that not all selection pressures shape the microbiome; indeed, in *Drosophila*, traits such as activity level, sleep, and some aspects of nutrition are known to not be influenced by the microbiome [39–42]. Our results suggest that the microbiome changes along with host evolution in the E&R context – although we emphasize that the data we present are a re-analysis of existing genomic data and not derived from new manipulative experiments. Our results here contribute to a growing body of literature suggesting that when the microbiome contributes to host phenotypic variation, changes in the microbiome have the potential to impact host evolutionary trajectories [6, 43]. The evolutionary interplay between host and their microbiome may play an important role in driving host evolution (beyond the explicit selective pressures exerted in these experiments), and this level of variation should not be ignored in E&R analyses.

We observed several generalities in the microbial response in E&R experiments. First, the microbiome in both control and evolved populations was composed of similar bacterial families (Fig. 1), suggesting selection did not lead to the complete replacement by different bacterial taxa in evolved populations. In evolved populations, only a few of the bacterial families increased in relative abundance. Furthermore, in all studies, replicate lines from both control and evolved populations show similar community compositions, suggesting consistent effects on the microbiome (Fig. 2, Supp. Figs. 1-10). Because community composition is consistent across replicates, we do not believe drift explains the observed reduction in diversity. If drift was a predominant force shaping the microbiome in evolved populations, we would have expected stochasticity to increase diversity across replicates within control and evolved populations. More likely, bacteria that contribute to the host adaptation may be more likely to persist under the selective pressure, increasing in abundance and facilitating local adaptation. Second, the increase in abundance of particular bacterial families, like *Wolbachia*, also contributed to the frequent reduction in diversity. The reduction in diversity likely reflects local adaptation in the microbiome, but potentially also the loss of genetic diversity in the host. We expect that the rapid nature of E&R experiments, combined with strong selective pressures, results in lower heterozygosity levels across the genome following selection in E&R experiments [13]. Host genetics shapes a significant fraction of the fly microbiome [44], and perhaps the loss of diversity in the host genome also contributed to the reduction in microbial diversity observed here. While evolved populations have reduced heterozygosity, they still maintain substantial heterozygosity across the genome. More research is necessary to understand how host genome-wide diversity affects microbial diversity, or if only certain host loci are the key drivers of changes in microbial diversity. Uncovering the specific host genetic loci that may be associated with microbial changes is beyond the scope of this current study; however this is an important factor to consider in future studies.

For the evolved microbiomes, bacteria may have evolved different functions that hosts can leverage. For example, the relative abundance of *Acetobacteraceae* is enriched in the evolved populations for accelerated development time (Fig. 1a). *Acetobacter* produces acetic acid that modulates the insulin/insulin-like signaling (IIS) growth factor pathway in flies [36]. More so, *Acetobacter* is frequently associated with accelerated development compared to other bacteria [24, 36, 45, 46]. The IIS pathway may also integrate metabolic products from other bacteria in the microbiome to help regulate fly metabolism. *Wolbachia* infection has been shown to increase insulin signaling in *Drosophila* [17]. The increased *Anaplasmataceae* abundance in the evolved populations may better regulate metabolic traits to mitigate selection in the starvation resistance experiment (Fig. 1h). We hypothesize that increased relative abundance for particular bacteria in the evolved populations corresponds to functional changes and is suggestive of fitness benefits for the fly. Subsequently, flies transmit and preferentially associate with the beneficial microbes. However, bacteria may also be increasing in the evolved conditions independently of any host selection. To better understand how microbial evolution interacts with host evolution, longitudinal sampling over the course of the evolutionary trajectory is necessary. Identifying if beneficial adaptations emerge first in the microbiome and then alter allele frequencies in host populations would provide key insights into how host-microbiome interactions shape eukaryotic evolutionary processes.

The temporal aspect of host-microbiome evolution is important, but underexplored and thus poorly understood. Our analysis suggests that time did not significantly affect the difference in diversity between control and evolved populations (Fig. 4). This might be because the microbiome changes rapidly, within a single host generation, but the evidence for rapid change is inconsistent. One study found that the microbiome was significantly different when flies were shifted to a high fat diet, but not when starved [47], while another study also found no differences when shifted to low or high sugar diets [48] within their lifespan. Finally, in flies mono-associated with *Lactobacillus* reared in nutrient poor diets, *Lactobacillus* evolved beneficial mutations that promoted fly growth in only 5 fly generations [49]. While most experiments did not manipulate diets in our E&R analysis, the findings from these three studies suggest a range of timescales in which the microbiome may evolve, though few studies have actually collected controlled time series data on microbiome change in *Drosophila*. Conducting longitudinal surveys of the microbiome during experimental evolution is essential to understanding if and how the microbiome shapes host evolutionary trajectories.

*Wolbachia* was found in most of the experiments and often increased in relative abundance in evolved populations (Fig. 5). *Wolbachia* has a variety of effects on fly biology, ranging from reproductive phenotypes to immunity to nutrition [50–52] and may substantially influence *Drosophila* evolution [50, 53]. The phenotypic effects exerted by *Wolbachia* on their hosts often depend on the degree of increase in abundance. For example, higher abundance provided stronger cytoplasmic incompatibilities [54], increased protection from viruses [55], or greater reductions in lifespan [56]. However, several factors may actually confound the *Wolbachia* results observed in our meta-analysis here. Infection was only assessed from pools of flies – we do not have access to individual level status (Table 1). Relative abundance may reflect the average relative abundance within individuals or heterogeneous infection patterns across individuals. We believe this second scenario of heterogeneous infection across individuals is not likely for the four high *Wolbachia* studies we examined in more detail. In a study that examined how *Wolbachia* infection spreads within outbred populations, *Wolbachia* infection across individuals increased to 80% by 10 generations and nearly 100% by 32 generations [57]. This suggests that for the four high *Wolbachia* studies, most individuals were infected. However, for the three studies with low *Wolbachia* abundance, it may be that *Wolbachia* infection status is highly heterogeneous across individuals or reflects recent *Wolbachia* infections (or simply contamination). Fly age also affects *Wolbachia* abundance, increasing in older flies [58]. Unfortunately, age of flies was only described in 3/10 studies (Table 1), but was always similar between control and evolved populations. Age might affect our results if control and evolved populations were systematically collected at different ages. We think this unlikely as it would also bias the genomic analysis as survival may differ between control and evolved populations at different ages (e.g., viability selection). Overall, designing experiments that explicitly control for *Wolbachia* infection is necessary to understand its potential influence on host-microbiome evolution [14, 23].

*Wolbachia* may also interact and change competitive interactions within the microbiome. In a comparison of a single genotype of flies infected and uninfected with *Wolbachia*, uninfected flies had twice as much *Acetobacter* [59]. However, in the same study, a different fly genotype did not display this effect. Yet, another study found that *Wolbachia* infection increased *Acetobacter* abundance [60]. These effects are inconsistent and likely depend on interactions between fly genotype, *Wolbachia* genotype, and environmental conditions. If *Wolbachia* interacts positively or negatively with different bacteria, then *Wolbachia* may also influence how the microbiome shapes host phenotypes and contributes to the host evolutionary trajectory.

*Wolbachia* may be more closely linked to the host evolutionary trajectory because it is vertically transmitted, while the rest of the *Drosophila* microbiome is environmentally acquired. The joint evolutionary trajectory with the host may change the response to selection in vertically transmitted microbes, like *Wolbachia*, compared to environmentally acquired microbes [5, 6]. Taken together, the interactions between *Wolbachia*, host, and microbiome are likely complicated. We note that computationally removing *Wolbachia* reads leads to differences in estimates for diversity, where sometimes diversity increases or decreases between control and evolved populations (Supp. Fig. 12). Manipulative experiments clearing *Wolbachia* infections and comparing the response in both the microbiome and host selection response would show if and how *Wolbachia* contributes to host evolution. ~ 50% of all arthropod species are predicted to be infected with *Wolbachia* or similar intracellular symbionts [61], and these microbe-microbe interactions may have important implications for the host [62].

While this is the first examination of the microbiome in the E&R context, other studies have implicated the microbiome in host adaptation in *D. melanogaster*. For example, as previously mentioned, when flies were monoassociated with *Lactobacillus plantarum* in nutrient poor environments, *L. plantarum* rapidly evolved symbiotic benefits to increase fly fitness [49]. Across replicates, the de novo appearance of several SNPs in the acetate kinase gene (*ackA*) in *L. plantarum* promoted larval growth and nutrition, and subsequently, this *L. plantarum* variant increased in frequency across fly generations. In another study, microbiome manipulation shifted allele frequency in seasonally evolving *D. melanogaster* to match latitudinal patterns of fly genetics [63]. Both of these studies rely on mono-associations with single microbes, but this likely does not realistically capture host-microbe dynamics. Higher order interactions among bacteria shape phenotypes in *Drosophila* [45, 46]. Interaction among microbes, like cross-feeding of metabolites between *Acetobacter* and *Lactobacillus*, can enable mutually beneficial growth for both bacteria species as well as increases bacterial growth, but critically also alters fly metabolism [64]. This suggests that mutations within bacterial species may affect interactions across bacteria in the microbiome. Furthermore, even strain-level variation within a bacterial species can have divergent effects on host phenotypes [65, 66]. The technical challenges associated with accurately quantifying genetic variation across complex microbial populations necessitated these mono-association experiments. Fortunately, new emerging methods are enabling the identification of signatures of selection in complex microbiomes [67, 68]. Future experiments with more complex and realistic microbiomes will show how microbe-microbe interactions contribute to host adaptation.

Taken together with our analyses, as the host evolves, the microbiome frequently changes in response to host selection. More generally, other systems like *Brassica* and *Arabidopsis* have also shown that selection on hosts changes the microbiome as well [69, 70]. In both these studies, transplanting an evolved microbiome into unevolved hosts changed host phenotypes, suggesting that the microbiome has the capacity to transfer adaptive potential. Similar approaches could be applied to *Drosophila* following E&R experiments. Importantly, our study here only characterized change in microbial community composition, but not how specific bacteria evolved (e.g., mutations or polymorphisms) in response to host selection. New computational and sequencing techniques that enable variant discovery in bacteria combined with longitudinal sampling have quantified eco-evolutionary dynamics in mammalian microbiomes to show that bacteria frequently acquire new mutations to increase fitness to respond to biotic fluctuations in the gut environment [71, 72]. Combined with the rich genetic resources and experimental ease in *Drosophila*, microbiome transplants and novel computation techniques will illuminate key processes underlying host-microbiome evolution.

We note the experiments analyzed here were not designed explicitly to test the role of the microbiome in host adaptation. This may impact our results in several ways. None of these studies were executed with quality control measures that can affect estimates of microbial diversity, such as process blanks during DNA extraction, no template controls during PCR, and batch effects during library preparation [67, 73–75]. While we applied an arbitrary cutoff to remove contaminants, it is difficult to know how potential contaminants may affect the observed results. However, contamination would have to differentially affect control and evolved microbiomes to influence our results--which we believe is unlikely. Surveys of microbial diversity in *D. melanogaster* typically use 16S rRNA profiling and find bacteria from the *Acetobacteraceae*, *Firmicutes*, and *Enterobacteriaceae* [22, 24, 76]. Our mapping approach detected these bacteria commonly associated with *D. melanogaster*, but also found abundant methanogens and human commensal microbes (Fig. 1). One discrepancy could arise from our metagenomic approach, which will often lead to different conclusions than 16S rRNA profiling [67]. Mining metagenomes from existing whole genome sequencing is an emerging area of research in the microbiome, and more work is necessary for biological interpretations [5, 67]. Finally, none of the flies sequenced in these studies were surface sterilized, and thus, the metagenomes characterized here result from both the external body surface and internal gut microbiome. However, the external microbiome is orders of magnitude less abundant than the internal microbiome across the fly lifespan [77]. While we cannot distinguish between external and internal microbiomes in this analysis, future studies should be clear if the total (external and internal) or gut microbiomes were sequenced. Nevertheless, the consistent differences in the microbiome across experiments shown here highlight how E&R experiments could provide exemplary opportunities to investigate the genetic basis underlying host-microbiome evolution.

## Conclusions

For researchers interested in adapting the E&R approach for host-microbiome interactions, we have several key recommendations. As we have suggested above, more intensive temporal sampling to capture both microbial and host evolution is necessary. For *Drosophila*-microbiome E&R experiments, researchers may wish to begin the experimental evolution by standardizing the microbiome between control and evolved populations, like with the 5-species bacterial community commonly used [23, 78] or fly feces to mimic natural, but standardized microbial inoculation [79, 80]. Second, as much of the microbiome is determined by the environment in flies, researchers need to use consistent brands of yeast, preservatives, and other aspects of diet/environment. For example, different preservatives have different effects on the microbiome and behavioral traits [40, 41]. *Drosophila* in different labs in the same building (with the same kitchen for fly food) had different microbiomes [76], suggesting that several aspects of the environment are important in shaping the fly microbiome. Finally, as outlined by Goodrich et al. [81], microbiome research requires careful planning (with both biological and technical controls), extensive documentation, and consistency. Importantly, we are not advocating that every E&R experiment incorporates the microbiome, but note that the microbiome may impact conclusions from E&R experiments. For researchers not explicitly interested in the microbiome, our primary recommendation is to clear fly lines of *Wolbachia* to avoid potential confounding effects between host genetic and *Wolbachia* evolution as others have suggested [14, 23].

In conclusion, the microbiome frequently responded to selection in ten E&R studies in *D. melanogaster.* Our results here associate the microbiome in the host response to some selective pressures, but more work is necessary to partition the relative effects of host genetics and microbial evolution. We observed large differences in bacterial diversity between control and evolved populations, but a key question remains--if and how the microbiome alters the host response to selection. Combining E&R experiments with approaches from quantitative genetics will be especially fruitful to dissecting the microbial contribution to host evolution [6]. Tracking the rate of microbial evolution over multiple timepoints during fly adaptation will be particularly helpful to elucidate whether the microbiome shapes the host evolutionary trajectory. Partitioning the microbial effects on host phenotype during adaptation may show that microbiome facilitates or impedes host adaptation. Reciprocal transplants over the course of host adaptation will also demonstrate how the microbiome modifies host evolution. Our results here suggest that the microbiome might influence host evolution, but do not prove it. To measure how the microbiome affects host evolutionary trajectory, combining several of these techniques will be necessary. Overall, incorporating the microbiome into E&R experiments will provide fundamental insights into host-microbiome evolution.

## Methods

We searched the literature for E&R experiments in *D. melanogaster* where replicated control and selection lines were derived from outbred populations and raw .fastq data were publicly available*.* We found 10 studies that met these criteria. Our analyses captured a wide range of different selection pressures, from life-history traits to abiotic and pathogen pressures, enabling generalizations about the microbiome response to host selection*.* In all cases, the E&R approach sequenced pools of individuals from different selection regimes, but each E&R study had different levels of replication (summarized in Table 1). We report the diet as described in the publication for each study (Table 1). While most studies did not publish specifics about the diet, we noted the diets that differed between control and evolved populations (only one study [31]); if the publication did not specify, we reasonably assumed diets were the same. These were the only data available from published E&R experiments in *D. melanogaster* at the time of submission.

Raw sequences were cleaned using Trimmomatic [82] to remove sequencing adapters, remove low quality reads (average quality per base > 15), and drop reads shorter than 20 bases long. Then, bacterial reads were assigned at the family level using Kraken [83]. Relative abundance of bacterial families were determined using Bracken [84]. We removed any low abundance bacterial family that was assigned fewer than 100 reads as potential contaminants.

Bacterial data was analyzed using the phyloseq package [85]. To assess if bacterial communities were fully sampled, rarefaction was performed (step size =1000) using ggrare [86]. Rarefaction curves indicate communities were fully sampled in all experiments (Supp. Figs 1-10). Beta-diversity to test differences in community composition between control and evolved populations was performed using PERMANOVA on Jaccard similarity. Bacterial alpha-diversity was calculated using the Shannon diversity index. For each experiment, each line was subsampled with replacement to the minimum number of reads in the experiment. Diversity was calculated on this rarefied library. The subsampling was performed 100 times to minimize stochasticity and artificial inflation of diversity associated with rarefaction [87]. Diversity was then averaged across the 100 subsampling efforts and compared between control and evolved lines. We determined significance using Welch’s t-test. We then tested whether two factors were sufficient to explain variation in microbial diversity between control and evolved lines: duration of selection (i.e., the number of fly generations) and *Wolbachia* infection.

First, the duration of selection ranged from 5 to 605 generations. We reasoned that selection response in the microbiome might be influenced by length of selection (the longer the selection, the more divergent the microbiome between control and evolved lines). To test if the duration of selection was correlated with changes in microbial diversity, we first calculated the average microbial diversity for the control lines. We then subtracted the diversity of each evolved line from the averaged control diversity to calculate change in diversity. Because we had positive and negative changes in diversity, we used the absolute difference. We performed a linear regression between change in diversity and the log10 duration of selection, modeled as Y = a + b + e, where Y = change in diversity, a = log10 duration of selection, b = random effect of experiment, and e = residual error. Lme4 was used to perform the regression in R [88].

Given that *Wolbachia* reads frequently make up the majority of the microbial reads (Supp. Table 1), we examined if *Wolbachia* relative abundance differed between control and evolved populations. We focused on only the four studies with *Wolbachia* relative abundance > 2%. Statistical significance was assessed with a Kruskal-Wallis test on Wolbachia relative abundance.

## Supplementary Information

**Additional file 1: Supp. Table 1**. Relative abundance of Wolbachia in each of the 10 E&R experiments

**Additional file 2: Supp. Table 2**. List of accession numbers for raw genomic data from the 10 E&R experiments

**Additional file 3: Supp. Table 3**. Statistical differences between control and evolved microbiomes with *Wolbachia* reads computationally removed

**Additional file 4: Supp. Figures 1-10**. Distribution of sequencing depth, rarefaction curves, and relative abundance of bacteria (family-level) for each of the 10 E&R experiments

**Additional file 5: Supp. Figure 11**. Estimates of random effects (i.e., E&R experiment) from linear model to test the correlation between microbial change and duration of selection

**Additional file 6: Supp. Fig. 12**. Differences in bacterial diversity when including (full bacterial community) or excluding *Wolbachia* reads in four of the E&R experiments analyzed

## Data Availability

The raw .fastq data are available in the NCBI Sequence Read Archive, and accession numbers can be found in Supp. Table 2. The code used to analyze data can be found at https://github.com/lphenry/extgeno_e-r.

## Abbreviations

E&R: Evolve and Resequence
IIS: Insulin/insulin-like signalling
