# Supplementary material for: Meta-analysis suggests the microbiome responds to Evolve and Resequence experiments in Drosophila melanogaster
Source: BMC Microbiol. 2021 Apr 9;21:108. doi: 10.1186/s12866-021-02168-4 (PMC8034159; doi:10.1186/s12866-021-02168-4)
Supplement: Supplementary file 1 — Additional file 1: Supp. Table 1. Relative abundance of Wolbachia in each of the 10 E&R experiments [file 12866_2021_2168_MOESM1_ESM.pdf]

Supp. Table 1: Relative abundance of Wolbachia in each experiment. For each experiment, the minimum, max, and average percentage of Anaplasmataceae reads is presented for both control (C) and evolved (E) lines, as well as total average for the experiment.

Study	C-Wolb avg	C-Wolb min	C-Wolb max	E-Wolb avg	E-Wolb min	E-Wolb max	Average
Accelerated development	0.02	0.02	0.03	1.32	1.24	1.41	0.67
Delayed reproduction	0.00	0.00	0.00	0.00	0.00	0.00	0.00
Increased lifespan	0.00	0.00	0.00	0.00	0.00	0.00	0.00
Egg size	0.03	0.03	0.05	0.06	0.03	0.12	0.05
Desiccation resistance	76.86	70.21	81.53	88.86	88.43	89.72	82.86
Fluctuating temps	62.51	48.69	75.35	72.22	68.45	75.15	65.42
Salt + cadmium resist	0.01	0.01	0.01	0.01	0.01	0.03	0.01
Starvation resistance	56.21	53.03	58.22	75.48	72.30	78.37	65.85
Parasitoid resistance	0.00	0.00	0.00	0.00	0.00	0.00	0.00
Viral resistance	96.39	95.20	97.31	97.72	97.57	98.09	96.84
