# Supplementary material for: Meta-analysis suggests the microbiome responds to Evolve and Resequence experiments in Drosophila melanogaster
Source: BMC Microbiol. 2021 Apr 9;21:108. doi: 10.1186/s12866-021-02168-4 (PMC8034159; doi:10.1186/s12866-021-02168-4)
Supplement: Supplementary file 2 — Additional file 2: Supp. Table 2. List of accession numbers for raw genomic data from the 10 E&R experiments [file 12866_2021_2168_MOESM2_ESM.pdf]

Supp. Table 2: Accession numbers for E&R experiments

Experiment	*Citation*	*Accession*
Accelerated development time	Burke Nature 2010	SRP002024
Delayed reproduction	Remolina Evolution 2012	SRA038471
Increased lifespan	Michalak BMC Genomics 2017	SRP092765
Egg size	Jha MBE 2015	PRJNA281953
Desiccation resistance	Kang BMC Genomics 2016	SRP066877
Fluctuating temperature	Orozco-terWengel Molecular Ecology 2012	ERP001290
Salt + cadmium resistance	Huang PLOS Genetics 2014	PRJNA257179
Starvation resistance	Hardy MBE 2018	PRJNA315172
Parasitoid resistance	Jalvingh PRSB 2014	PRJNA170455
Viral resistance	Martins PNAS 2014	ERX409654
