# Supplementary material for: Meta-analysis suggests the microbiome responds to Evolve and Resequence experiments in Drosophila melanogaster
Source: BMC Microbiol. 2021 Apr 9;21:108. doi: 10.1186/s12866-021-02168-4 (PMC8034159; doi:10.1186/s12866-021-02168-4)
Supplement: Supplementary file 3 — Additional file 3: Supp. Table 3. Statistical differences between control and evolved microbiomes with Wolbachia reads computationally removed [file 12866_2021_2168_MOESM3_ESM.pdf]

Supp. Table 3: Statistical differences between control and evolved microbiomes with *Wolbachia* reads computationally removed.

**Pressure**	**test stat**	**df**	**significance**
accelerated development without *Wolbachia*	t = -5.511	df = 3.052	p-value = 0.011
egg size without *Wolbachia*	t = 0.250	df = 3.077	p-value = 0.818
desiccation resistance without *Wolbachia*	t = 0.394	df = 3.985	p-value = 0.714
fluctuating temperature without *Wolbachia*	t = -4.135	df = 6.886	p-value = 0.005
salt and cadmium resistance without *Wolbachia*	t = 1.431	df = 2.287	p-value = 0.274
starvation resistance without *Wolbachia*	t = -1.413	df = 2.903	p-value = 0.255
viral resistance without *Wolbachia*	t = -2.851	df = 7.193	p-value = 0.024
