# Supplementary material for: Meta-analysis suggests the microbiome responds to Evolve and Resequence experiments in Drosophila melanogaster
Source: BMC Microbiol. 2021 Apr 9;21:108. doi: 10.1186/s12866-021-02168-4 (PMC8034159; doi:10.1186/s12866-021-02168-4)
Supplement: Supplementary file 4 — Additional file 4: Supp. Figures 1-10. Distribution of sequencing depth, rarefaction curves, and relative abundance of bacteria (family-level) for each of the 10 E&R experiments [file 12866_2021_2168_MOESM4_ESM.pdf]

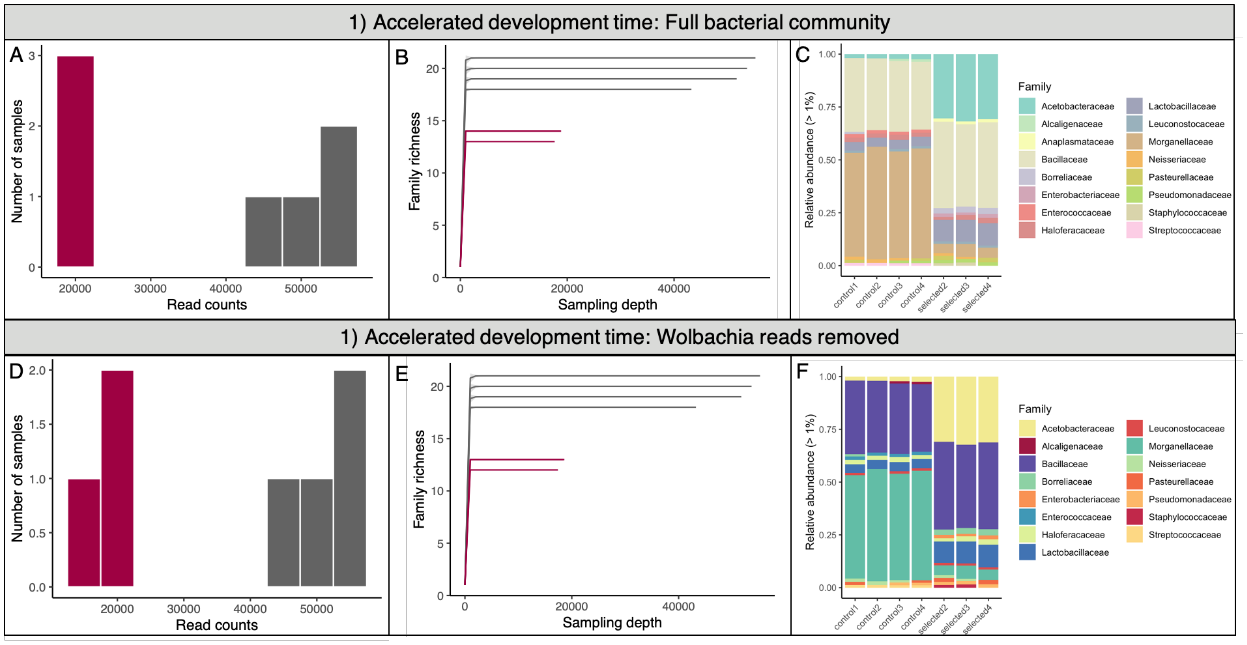

Supp. Fig. 1: Accelerated development time. The control population is in grey, while dark red denotes the evolved populations. A) Histogram of sequencing depth for the seven pools in this study. B) Rarefaction curves suggest that bacterial communities were fully sampled, even though sequencing depth was lower for the evolved populations. C) Relative abundance of each pool shows bacterial taxa with each color. D-F) Histogram of sequencing depth, rarefaction, and relative abundance of bacterial families following removal of *Wolbachia* reads.

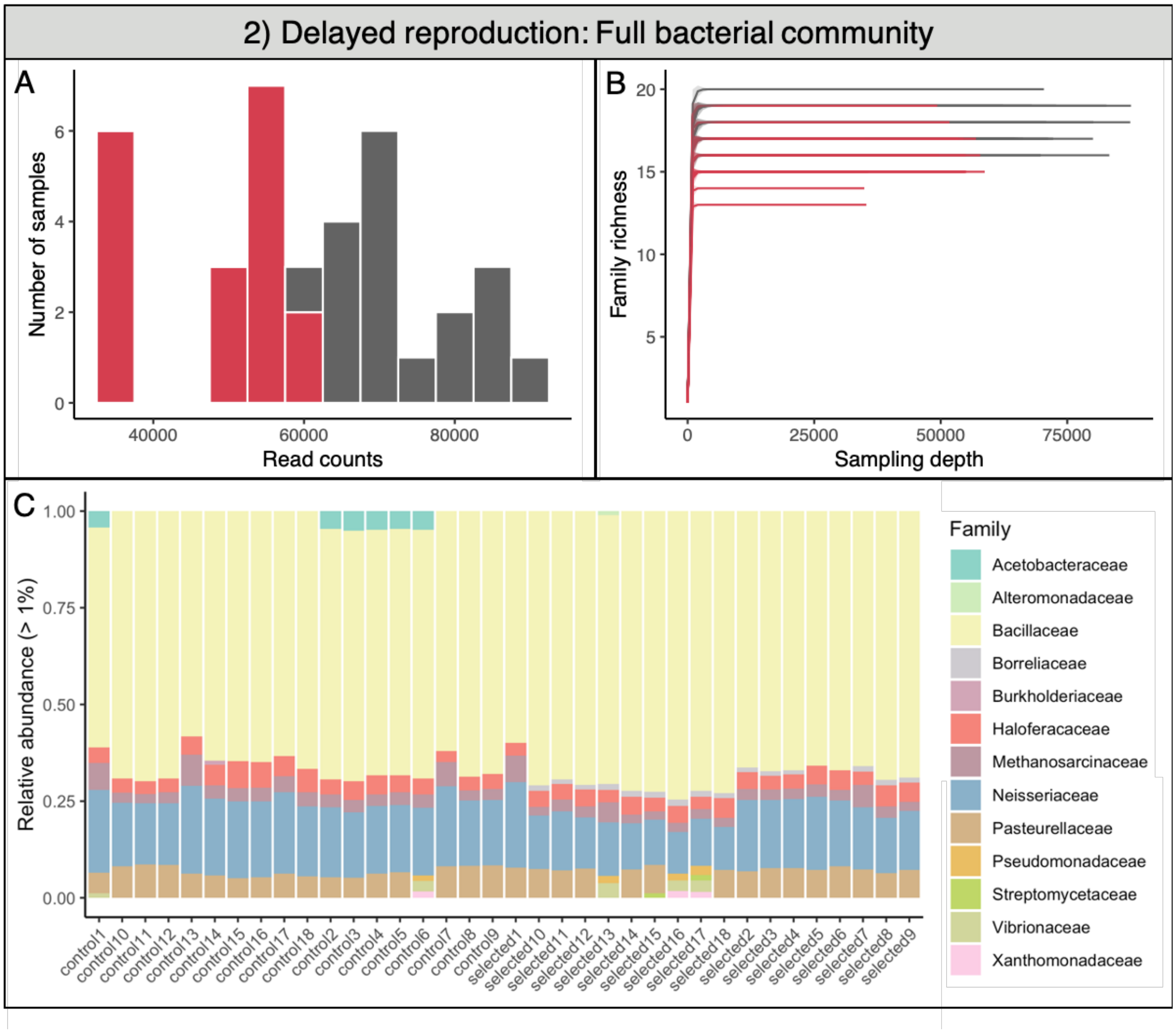

Supp. Fig. 2: Delayed reproduction. The control population is in grey, while red denotes the evolved populations. A) Histogram of sequencing depth for the 36 pools in this study. B) Rarefaction curves suggest that bacterial communities were fully sampled, even though sequencing depth tended to be lower for the evolved populations. C) Relative abundance of each pool shows bacterial taxa with each color. *Wolbachia* was not present in this study.

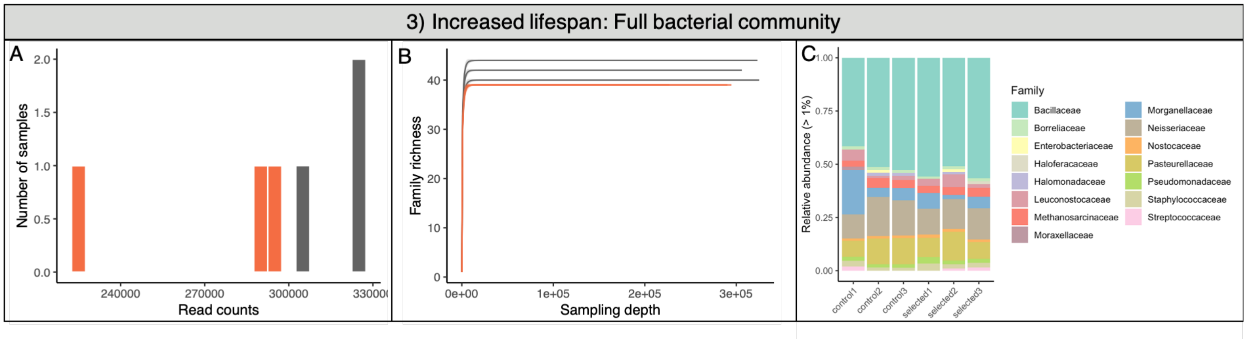

Supp. Fig. 3: Increased lifespan. The control population is in grey, while dark orange denotes the evolved populations. A) Histogram of sequencing depth for the six pools in this study. B) Rarefaction curves suggest that bacterial communities were fully sampled, even though sequencing depth was lower for the evolved populations. C) Relative abundance of each pool shows bacterial taxa with each color. *Wolbachia* was not present in this study.

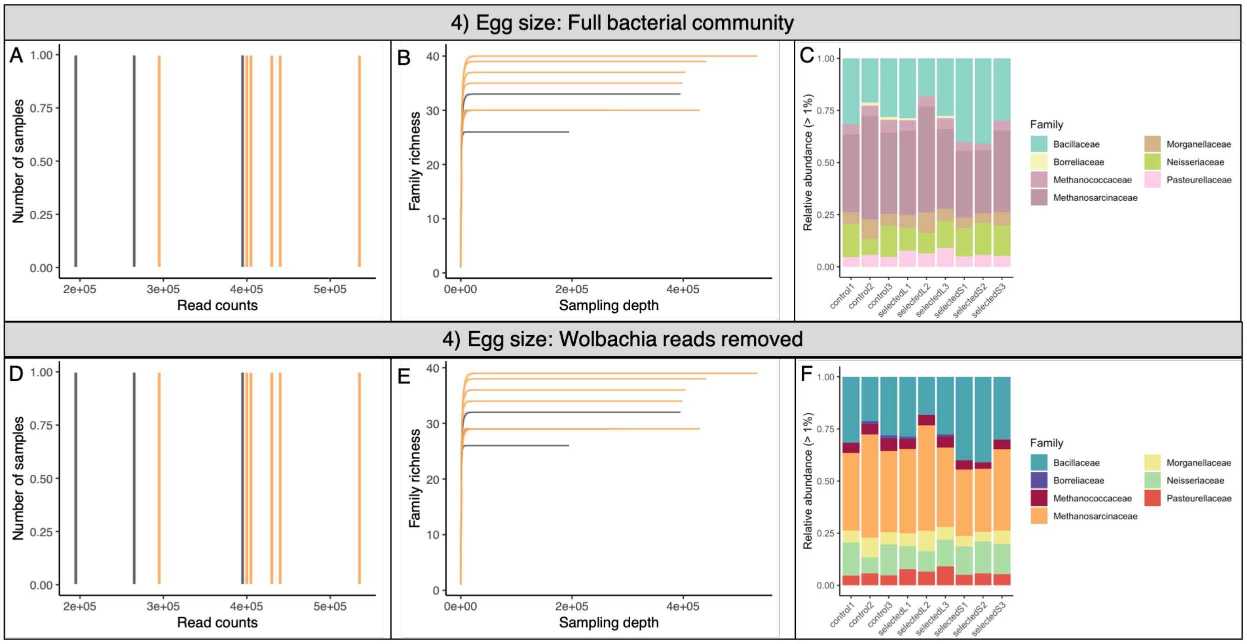

Supp. Fig. 4: Egg size. The control population is in grey, while orange denotes the evolved populations. A) Histogram of sequencing depth for the nine pools in this study. B) Rarefaction curves suggest that bacterial communities were fully sampled, even though sequencing depth was tended to be lower for the control populations. C) Relative abundance of each pool shows bacterial taxa with each color. D-F) Histogram of sequencing depth, rarefaction, and relative abundance of bacterial families following removal of *Wolbachia* reads.

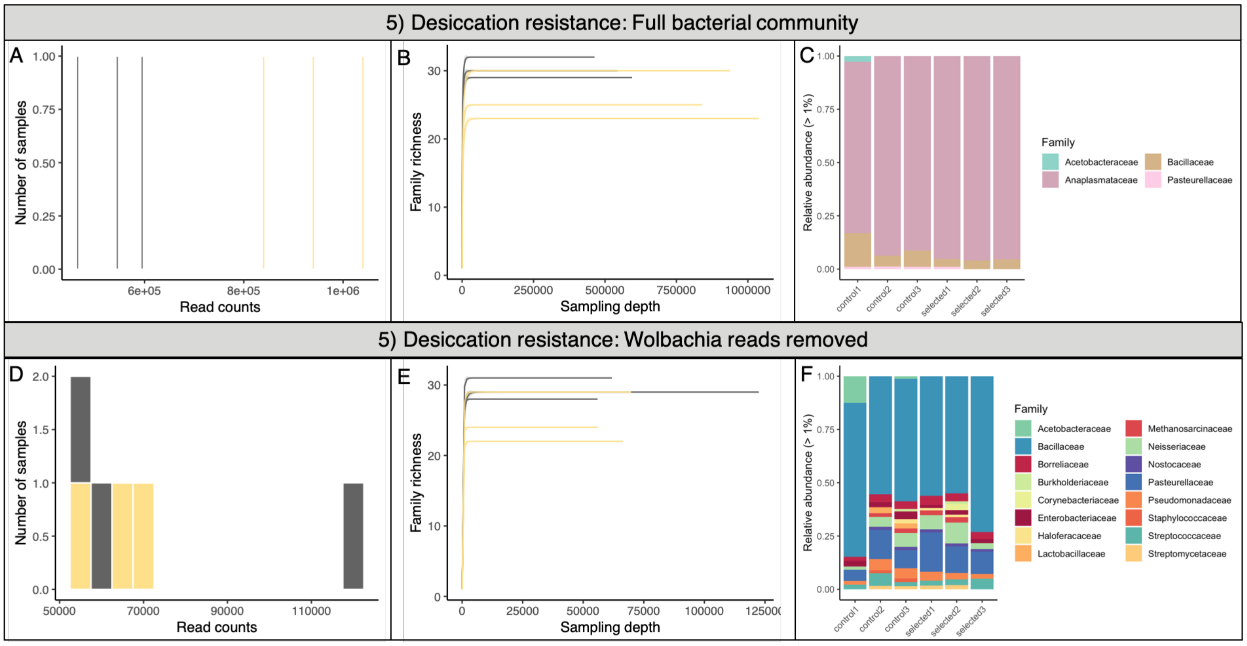

Supp. Fig. 5: Desiccation resistance. The control population is in grey, while dark yellow denotes the evolved populations. A) Histogram of sequencing depth for the six pools in this study. B) Rarefaction curves suggest that bacterial communities were fully sampled. C) Relative abundance of each pool shows bacterial taxa with each color. D-F) Histogram of sequencing depth, rarefaction, and relative abundance of bacterial families following removal of *Wolbachia* reads.

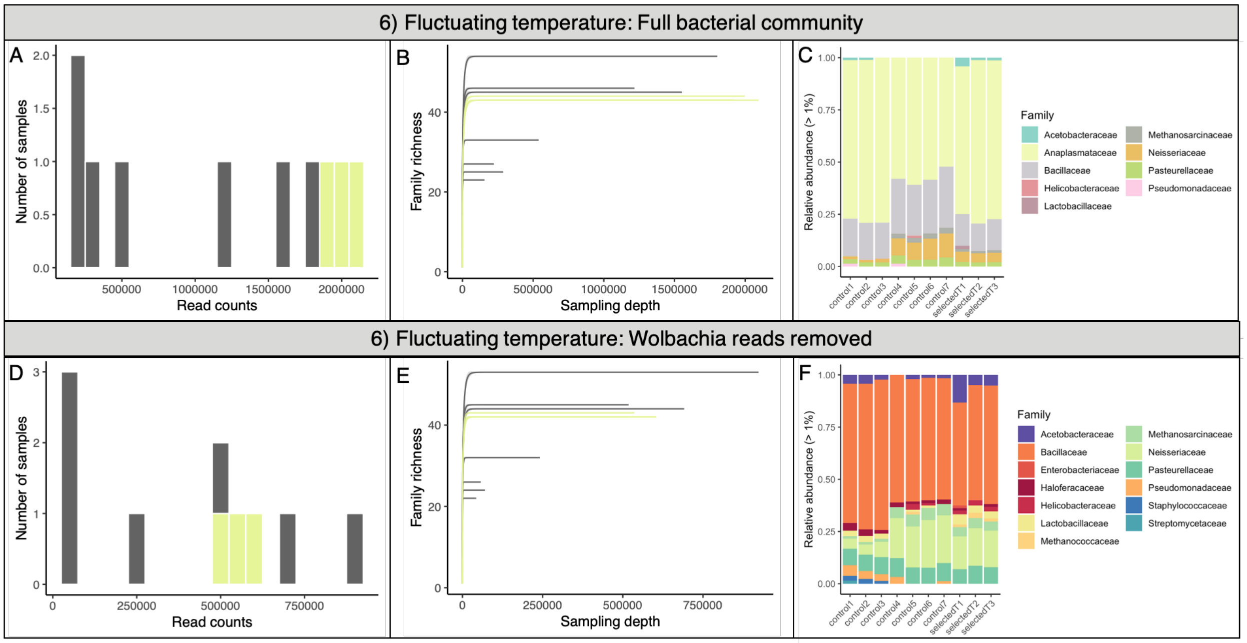

Supp. Fig. 6: Fluctuating temperature. The control population is in grey, while yellow denotes the evolved populations. A) Histogram of sequencing depth for the 10 pools in this study. B) Rarefaction curves suggest that bacterial communities were fully sampled, even though sequencing depth was lower for the control populations. C) Relative abundance of each pool shows bacterial taxa with each color. D-F) Histogram of sequencing depth, rarefaction, and relative abundance of bacterial families following removal of *Wolbachia* reads.

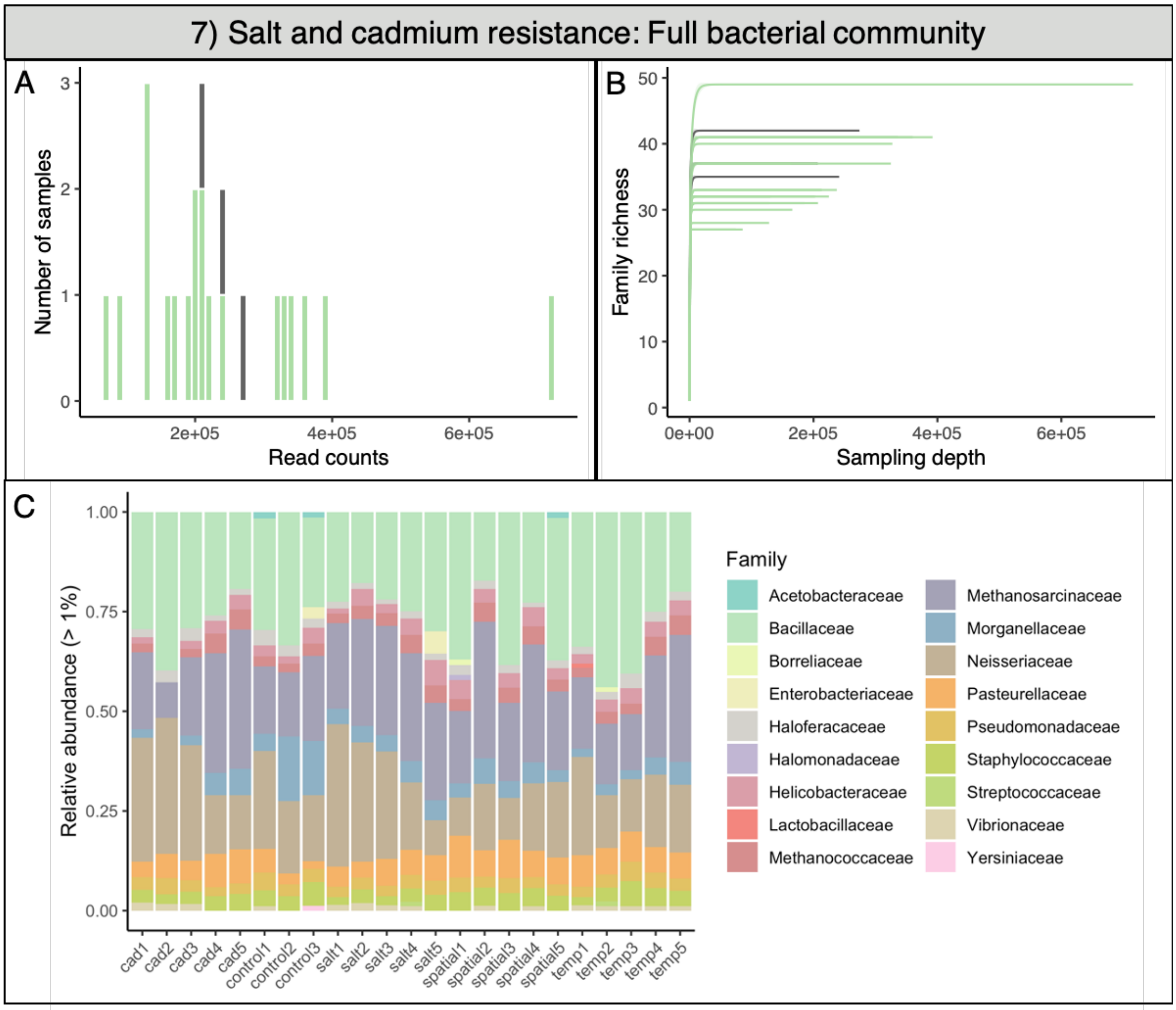

Supp. Fig. 7: Salt and cadmium resistance. The control population is in grey, while green denotes the evolved populations. A) Histogram of sequencing depth for the 23 pools in this study. B) Rarefaction curves suggest that bacterial communities were fully sampled, even though sequencing depth was lower for the evolved populations. C) Relative abundance of each pool shows bacterial taxa with each color. While *Wolbachia* was present in this study, it was below 1% relative abundance.

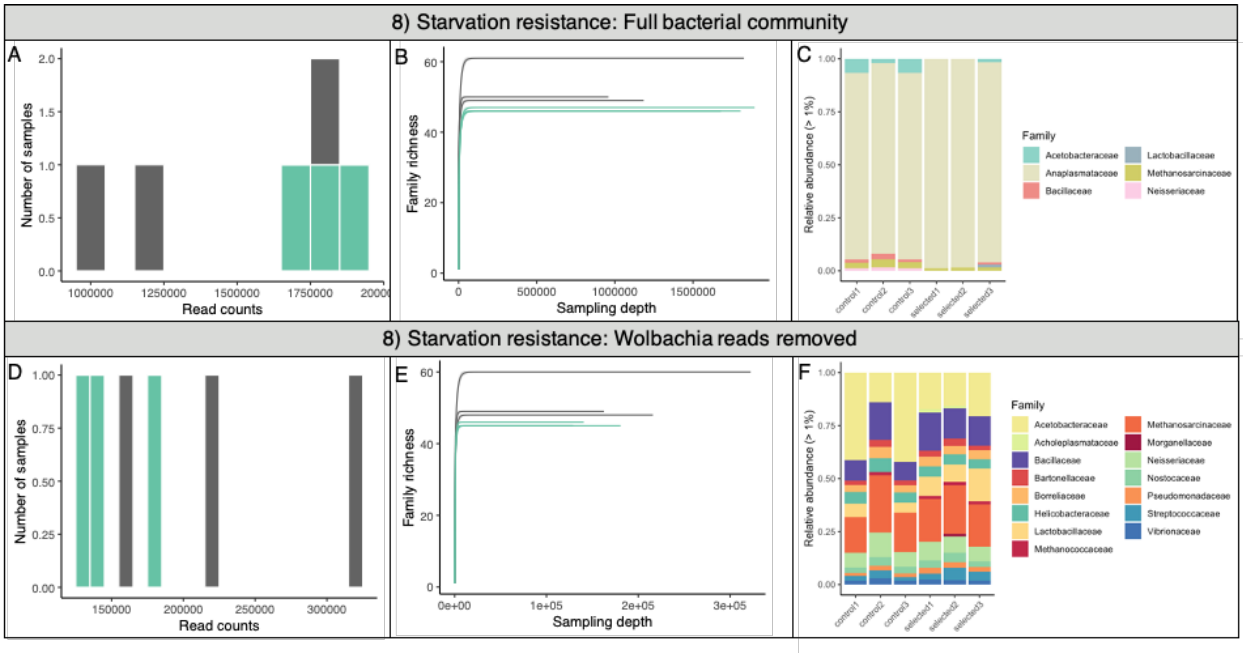

Supp. Fig. 8: Starvation resistance. The control population is in grey, while teal denotes the evolved populations. A) Histogram of sequencing depth for the six pools in this study. B) Rarefaction curves suggest that bacterial communities were fully sampled. C) Relative abundance of each pool shows bacterial taxa with each color. D-F) Histogram of sequencing depth, rarefaction, and relative abundance of bacterial families following removal of *Wolbachia* reads.

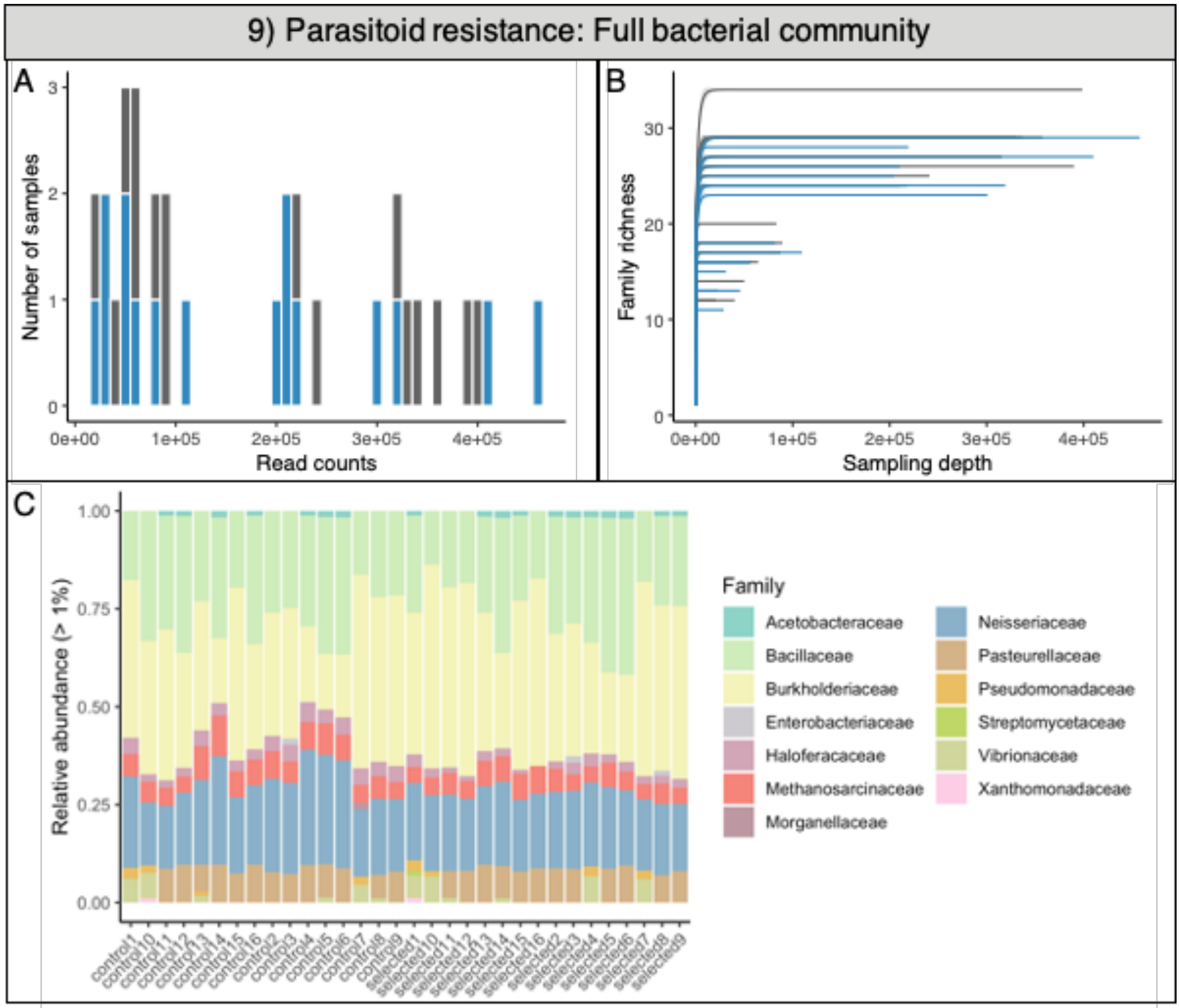

Supp. Fig. 9: Parasitoid resistance. The control population is in grey, while blue denotes the evolved populations. A) Histogram of sequencing depth for the 24 pools in this study. B) Rarefaction curves suggest that bacterial communities were fully sampled. C) Relative abundance of each pool shows bacterial taxa with each color. *Wolbachia* was not present in this study.

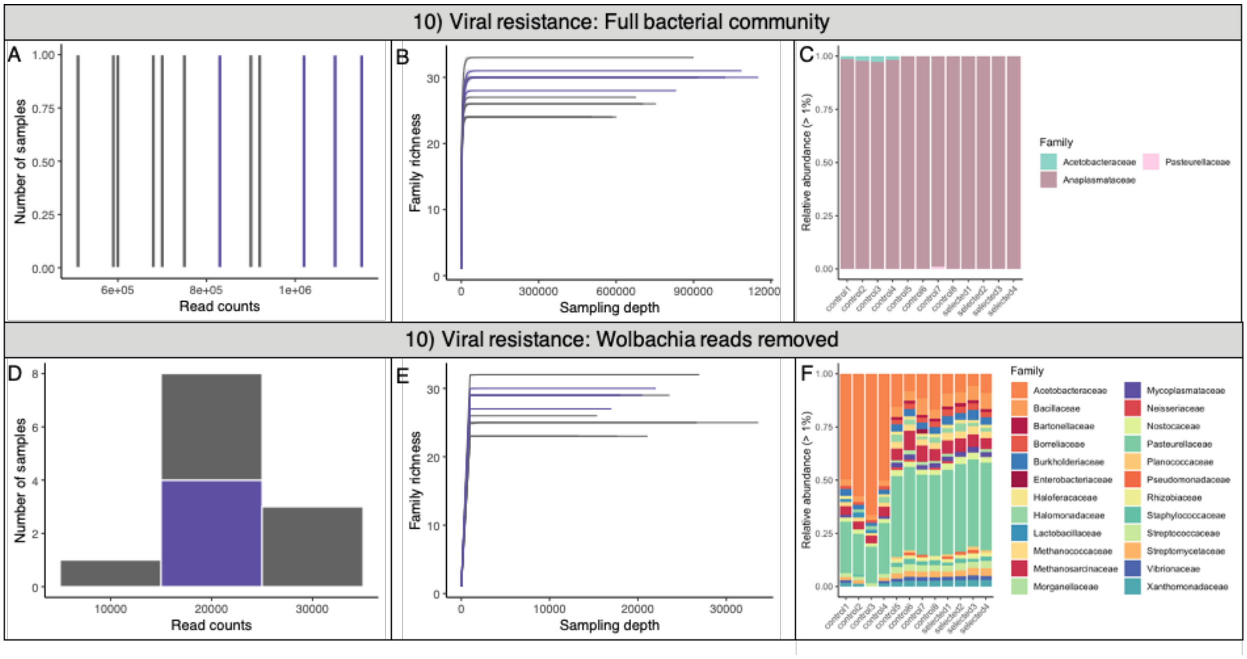

Supp. Fig. 10: Viral resistance. The control population is in grey, while purple denotes the evolved populations. A) Histogram of sequencing depth for the 12 pools in this study. B) Rarefaction curves suggest that bacterial communities were fully sampled. C) Relative abundance of each pool shows bacterial taxa with each color. D-F) Histogram of sequencing depth, rarefaction, and relative abundance of bacterial families following removal of *Wolbachia* reads.
