# Supplementary material for: Meta-analysis suggests the microbiome responds to Evolve and Resequence experiments in Drosophila melanogaster
Source: BMC Microbiol. 2021 Apr 9;21:108. doi: 10.1186/s12866-021-02168-4 (PMC8034159; doi:10.1186/s12866-021-02168-4)
Supplement: Supplementary file 5 — Additional file 5: Supp. Figure 11. Estimates of random effects (i.e., E&R experiment) from linear model to test the correlation between microbial change and duration of selection [file 12866_2021_2168_MOESM5_ESM.pdf]

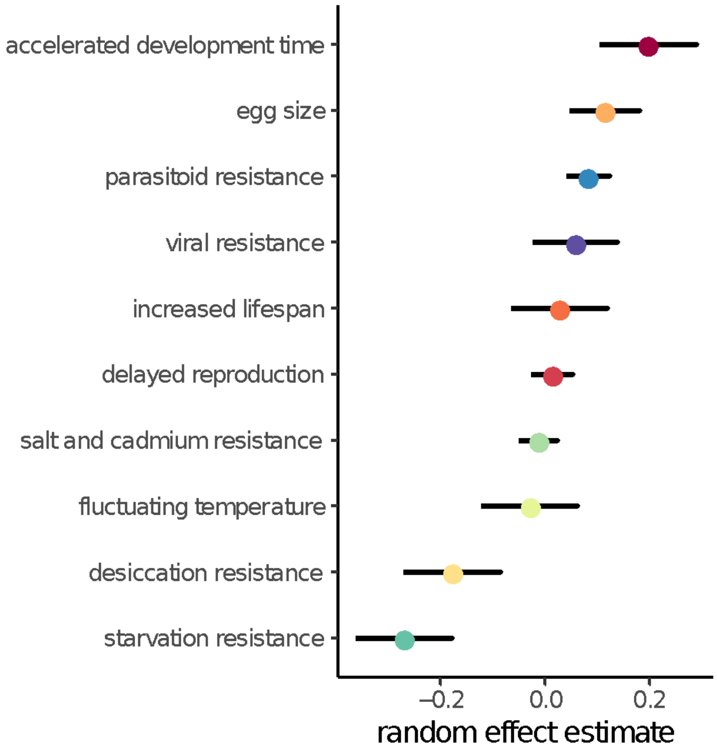

Supp. Fig. 11: Estimates of random effects for each experiment. Points show conditional means for the random effects and lines show +/- 2 conditional SD.
