# Supplementary material for: Meta-analysis suggests the microbiome responds to Evolve and Resequence experiments in Drosophila melanogaster
Source: BMC Microbiol. 2021 Apr 9;21:108. doi: 10.1186/s12866-021-02168-4 (PMC8034159; doi:10.1186/s12866-021-02168-4)
Supplement: Supplementary file 6 — Additional file 6: Supp. Fig. 12. Differences in bacterial diversity when including (full bacterial community) or excluding Wolbachia reads in four of the E&R experiments analyzed [file 12866_2021_2168_MOESM6_ESM.pdf]

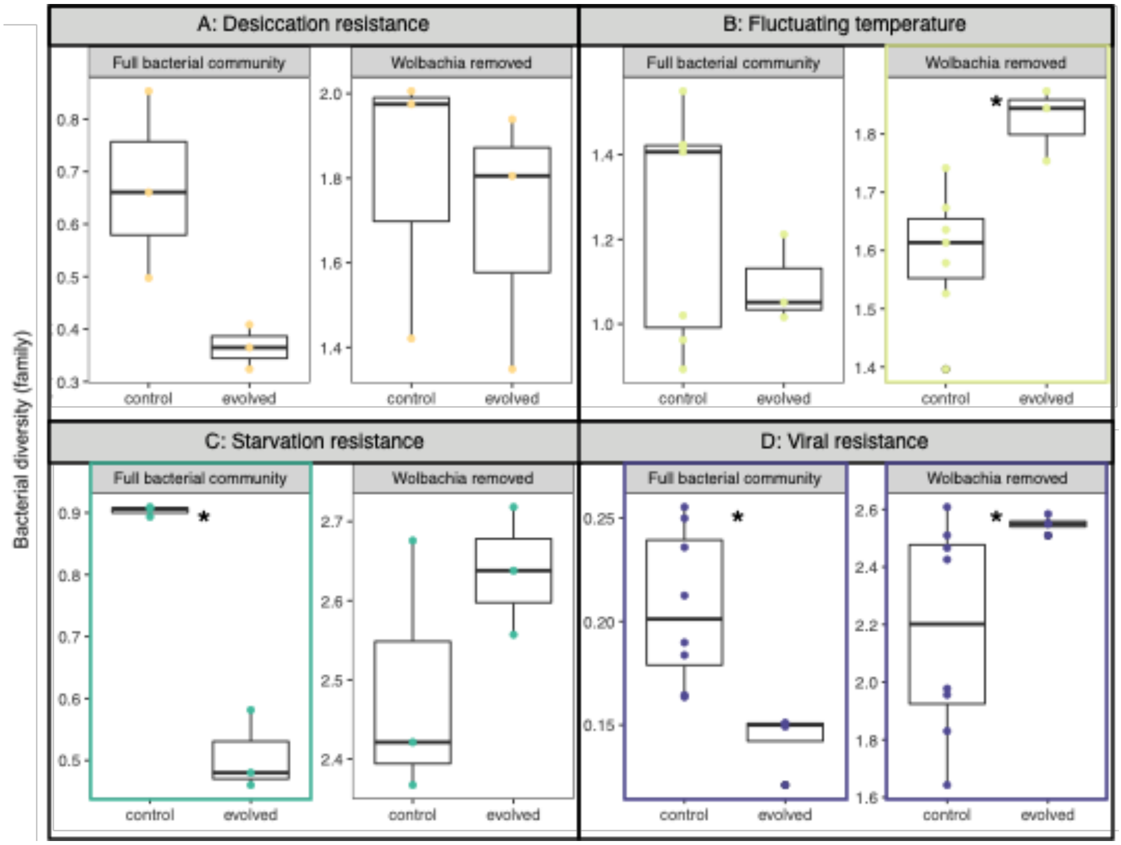

Supp. Fig. 12: Differences in bacterial diversity when including (full bacterial community) or excluding *Wolbachia* reads in four of the E&R experiments analyzed. Each point represents bacterial diversity (Shannon index) for a pool of sequenced flies. Scale bars are different because diversity comparisons are within either the full community or *Wolbachia* removed groupings for each experiment. We removed the Anaplasmataceae family (all of which are *Wolbachia* in flies and subsampled with replacement to the minimum number of reads in each experiment. Then, Shannon diversity was calculated on the rarified libraries as described in the methods in the main text.

The four experiments highlight how the difference between control and evolved microbiomes depends on whether *Wolbachia* is included. For example, for starvation resistance, diversity for the whole community is reduced when *Wolbachia* is included. However, when *Wolbachia* is excluded, there is no response in bacterial diversity between control and evolved populations. Full summary of differences can be found in Supp. Table 3.

While these results are not surprising given that a large number of reads were removed (though following *Wolbachia* removed, rarefaction curves suggest communities were still fully sampled as in Supp Fig. 1-10), the directionality of effect is surprising. We highlight these results because there is ambiguity about including Wolbachia in the microbiome. Many studies in *D. melanogaster* either use uninfected flies [1–4] or computationally remove reads during 16S rRNA microbiome analysis [5–9]. Furthermore, while 7/10 studies used flies infected with *Wolbachia*, only two studies explicitly mentioned *Wolbachia* infection in their flies--fluctuating temperature and viral resistance. As discussed in the main text, several studies suggest that

Wolbachia antagonistically interacts with Acetobacter. These data of course cannot answer the question if *Wolbachia* affects the microbiome--rather our analysis highlights the need for additional studies that explicitly manipulate Wolbachia infection status and measures both the response of the microbiome and fly during experimental evolution.

REFERENCES

1. Newell PD, Douglas AE. Interspecies Interactions Determine the Impact of the Gut Microbiota on Nutrient Allocation in Drosophila melanogaster. Appl Environ Microbiol. 2013;80:788–96.

2. Gould AL, Zhang V, Lamberti L, Jones EW, Obadia B, Korasidis N, et al. Microbiome interactions shape host fitness. Proc Natl Acad Sci U S A. 2018;115:E11951–60.

3. Selkrig J, Mohammad F, Ng SH, Chua JY, Tumkaya T, Ho J, et al. The Drosophila microbiome has a limited influence on sleep, activity, and courtship behaviors. Sci Rep. 2018;8:10646.

4. Douglas AE. The Drosophila model for microbiome research. Lab Anim . 2018;47:157–64.

5. Staubach F, Baines JF, Künzel S, Bik EM, Petrov DA. Host species and environmental effects on bacterial communities associated with Drosophila in the laboratory and in the natural environment. PLoS One. 2013;8:e70749.

6. Adair KL, Wilson M, Bost A, Douglas AE. Microbial community assembly in wild populations of the fruit fly Drosophila melanogaster. ISME J. 2018;12:959–72.

7. Walters AW, Hughes RC, Call TB, Walker CJ, Wilcox H, Petersen SC, et al. The microbiota influences the Drosophila melanogaster life history strategy. Mol Ecol. 2019. doi:10.1111/mec.15344.

8. Rudman SM, Greenblum S, Hughes RC, Rajpurohit S, Kiratli O, Lowder DB, et al. Microbiome composition shapes rapid genomic adaptation of Drosophila melanogaster. Proc Natl Acad Sci U S A. 2019;116:20025–32.

9. Wang Y, Kapun M, Waidele L, Kuenzel S, Bergland A, Staubach F. Continent-wide structure of bacterial microbiomes of European Drosophila melanogaster suggests host-control. bioRxiv. 2019;:527531. doi:10.1101/527531.
